# High incidence of severe cyclosporine neurotoxicity in children affected by haemoglobinopaties undergoing myeloablative haematopoietic stem cell transplantation: early diagnosis and prompt intervention ameliorates neurological outcome

**DOI:** 10.1186/1824-7288-36-14

**Published:** 2010-02-06

**Authors:** Anna Noè, Barbara Cappelli, Alessandra Biffi, Robert Chiesa, Ilaria Frugnoli, Erika Biral, Valentina Finizio, Cristina Baldoli, Paolo Vezzulli, Fabio Minicucci, Giovanna Fanelli, Rossana Fiori, Fabio Ciceri, Maria Grazia Roncarolo, Sarah Marktel

**Affiliations:** 1 Pediatric Immunohaematology and Bone Marrow Transplantation Unit, San Raffaele Scientific Institute, Milan, Italy; 2 San Raffaele Telethon Institute for Gene Therapy (HSR-TIGET), Milan, Italy; 3 Deparment of Neuroradiology, San Raffaele Scientific Institute, Milan, Italy; 4 Deparment of Clinical Neurophysiology, San Raffaele Scientific Institute, Milan, Italy; 5 Department of Anesthesia and Intensive Care, San Raffaele Scientific Institute, Milan, Italy; 6 Haematology and Bone Marrow Transplantation Unit, San Raffaele Scientific Institute, Milan, Italy; 7 Vita-Salute San Raffaele University Medical School, Milan, Italy

## Abstract

**Background:**

Neurotoxicity is a recognized complication of cyclosporine A (CSA) treatment. The incidence of severe CSA-related neurological complications following hematopoietic stem cell transplantation (HSCT) is 4-11%.

**Methods:**

We describe 6 cases of CSA related neurotoxicity out of 67 matched related HSCT performed in paediatric Middle East patients affected by haemoglobinopaties (5 beta thalassemia major, 1 sickle cell disease-SCD). Conditioning regimen consisted of iv busulphan, cyclophosphamide and graft-versus-host-disease (GvHD) prophylaxis with CSA, methylprednisolone, methotrexate and ATG.

**Results:**

All 6 patients presented prodromes such as arterial hypertension, headache, visual disturbances and vomiting, one to two days before overt CSA neurotoxicity. CSA neurotoxicity consisted of generalized seizures, signs of endocranial hypertension and visual disturbances at a median day of onset of 11 days after HSCT (range +1 to +40). Brain magnetic resonance imaging (MRI) performed in all subjects showed reversible leukoencephalopathy predominantly in the posterior regions of the brain (PRES) in 5/6 patients. EEG performed in 5/6 patients was always abnormal. Neurotoxicity was not explainable by high CSA blood levels, as all patients had CSA in the therapeutic range with a median of 178 ng/ml (range 69-250). CSA was promptly stopped and switched to tacrolimus with disappearance of clinical and radiological findings. All patients are symptoms-free at a median follow up of 882 days (range 60-1065).

**Conclusions:**

Our experience suggests that paediatric patients with haemoglobinopaties have a high incidence of CSA related neurological events with no correlation between serum CSA levels and neurotoxicity. Prognosis is good following CSA removal. Specific prodromes such as arterial hypertension, headache or visual disturbances occurring in the early post-transplant period should be carefully evaluated with electrophysiological and MRI-based imaging in order to intervene promptly and avoid irreversible sequels.

## Background

Cyclosporin A (CSA) is a calcineurin inhibitor routinely used as graft-versus-host-disease (GvHD) prophylaxis after haematopoietic stem cell transplantation (HSCT). Common CSA related side-effects include renal dysfunction, arterial hypertension, hepatic toxicity, gingival hyperplasia, hypertrichosis, opportunistic infections and tremors[1]. Severe neurotoxicity such as confusion, disorientation, decreased responsiveness, visual hallucinations, delusions, seizures, pyramidal motor weakness, cortical blindness, aphasia and ataxia is reported in 4% to 11% patients undergoing HSCT [2-6]. Unfortunately neurotoxicity can occur both at therapeutic and at high CSA levels [2,3]. The reported incidence of CSA neurotoxicity post HSCT may be underestimated because the clinical picture could be confused with other neurological complications such as infections, metabolic disturbances (hypomagnesaemia, hypocolesterolaemia, hypo/hypernatraemia, hypercalcaemia), toxic encephalopathy (drug induced by intratecal or systemic chemotherapeutic agents, oppioids, amphotericin B or radiotherapy), thrombotic microagiopathy, vascular events and structural neurological lesions[4]. Many risk factors for the development of CSA-related neurotoxicity have been investigated, including the use of methylprednisolone, arterial hypertension, fluid overload, hypocholesterolaemia, hypomagnesaemia and pre-existing brain disease[5,7]. The brain magnetic resonance imaging (MRI) finding usually associated to CSA neurotoxicity is known as posterior reversible leukoencephalopathy syndrome (PRES), typically distributed in the posterior regions of the white matter of the brain [5-8]. In general the prognosis of CSA neurotoxicity is good[4] and posterior leukoencephalopathy usually resolves completely with dose reduction or drug withdrawal. However, some cases of non-reversible encephalopathy syndrome have been described with occurrence of late epilepsy and late persistent electroencephalogram (EEG) abnormalities [9,10].

We report 6 cases of CSA related neurotoxicity occurring among 67 children undergoing HSCT for haemoglobinopaties and discuss early diagnosis and management.

## Methods

### Patients' characteristics

A total of 67 pediatric patients affected by haemoglobinopaties underwent matched related HSCT at our Institute between June 2005 and Nov 2008. Children were referred to our center by Mediterranean and Middle East Countries. Cost of treatment was covered by a cooperation program funded by the Mediterranean Institute of Haematology http://www.fondazioneime.org. Among these, 6 patients (9%) had evidence of severe CSA related neurotoxicity requiring CSA discontinuation. The underlying disease was beta thalassemia major (n = 5) or sickle cell disease (SCD, n = 1). Patients' characteristics are shown in table 1. According to the risk classes described by Lucarelli[11], 3 thalassemia patients were classified as class II and 2 as class III. None of the patients had preexisting neurologic deficits. Baseline neurological involvement was excluded in the SCD patient by MRI and EEG. Conditioning regimen is summarized in table 2. All patients received seizures prophylaxis with oral clonazepam (0.05 mg/kg/day) starting 24 hours before the first busulfan dose to 24 hours after the last dose.

**Table 1:** Patient's characteristics

Patient number	#1	#2	#3	#4	#5	#6
**Age** (yrs)	14	9	12	8	11	17

**Gender** (M/F)	M	F	M	F	F	M

**Country of origin**	Egypt	Lebanon	Iraq	Syria	Syria	Syria

**Diagnosis (Class)**	SCD	B-thal (2)	B-thal (3)	B-thal (3)	B-thal (2)	B-thal (2)

**AST/ALT** (U/L) pre HSCT	69/18	44/12	65/73	64/79	62/68	20/33

**Ferritin** (ng/ml) pre HSCT	3344	1855	3003	4930	3422	1287

**Portal fibrosis** Ishak grading (G) and staging (S)	G1/S4	G1/S1	G7/S4	G5/S3	G3/S3	G1/S1

**Iron chelation**	irregular	regular	irregular	irregular	regular	irregular

**HCV status **(Ab/RNA)	HCVAb+ RNA -	HCVAb - RNA -	HCVAb+ RNA +	HCVAb - RNA -	HCVAb -RNA -	HCVAb+ RNA -

**Table 2:** Conditioning regimen

	Pre-conditioning (-45 to -12)	Conditioning (-11 to -2)
B-thal/Class 2	//	iv Bu^§ ^Cy 200 mg/kg

B-thal/Class 3	AZ/HU Fludarabine 150 mg/m^2^	iv Bu^§ ^Cy 160 mg/kg ATG 7.5 mg/kg

SCD	AZ/HU Fludarabine 150 mg/m^2^	iv Bu^§ ^Cy 200 mg/kg ATG 10 mg/kg

### CSA administration and monitoring

GvHD prophylaxis consisted of CSA, short course of iv Methotrexate (MTX) 10 mg/m2 on d 1, 3 and 6 and metilprednisolone 0.5 mg/kg from d -1 until d 25. ATG was added in 3 patients.

CSA was used orally at the dose of 10 mg/kg/day from day -2. The dose was adjusted to a trough plasma level of 150-250 ng/mL with twice weekly monitoring. In the absence of GvHD, CSA was tapered from d 60 until discontinuation at 1 year.

Monitoring for CSA related side effects consisted of daily examination, 6 hourly blood pressure and daily blood routine biochemistry including magnesium till day 30, followed by 3 times weekly till day 60. Arterial hypertension above 75° percentile for age and weight was treated with oral calcium antagonists. Fluid overload was treated with iv furosemide. Magnesium was supplemented for plasma levels below 0.8 mmol/Lt. Children developing CSA neurotoxicity underwent neuro-radiological (MRI scan) and electrophysiological studies (EEG). In case of epileptic signs, EEG was repeated after 24 hours and then on regular basis in order to monitor the outcome of the anticonvulsive therapy. CSA-related toxicity was evaluated using the National Cancer Institute common toxicity criteria (NCI/CTC). Clinical management was lead by the HSCT specialists on the HSCT ward with the collaboration of intensivists, neurologists, neurophysiologists and neuroradiologists.

## Results

### Clinical course

All six patients developing neurotoxicity presented prodromes 1-2 days before overt neurotoxicity. Prodromes were arterial hypertension (n = 5), headache (n = 2), visual disturbances (n = 1) or vomiting (n = 1). Symptoms of CSA neurological toxicity were characterized by: generalized seizures (grade 3-4 neurotoxicity) (n = 4), endocranial hypertension evidenced by impaired level of consciousness (grade 3 confusion) associated to bradycardia, arterial hypertension and cerebral vomiting (n = 1), and blurred vision (grade 3 visual toxicity) (n = 1). The median day of appearance was 11 days after HSCT (range +1 to +40). When neurological symptoms appeared, CSA plasma levels were in range in all patients (median 178, range 69-250 ng/ml). Clinical and laboratory findings are summarized in Table 3. None of the patients had evidence of thrombotic microangiopathy. None of the clinical pictures were suggestive for viral encephalitis, as evidenced by apyrexia, normal CRP, negative molecular virological screening on plasma, non suggestive MRI and EEG pictures.

**Table 3:** Characteristics and treatment of CSA related neurotoxicity

Patient number	#1	#2	#3	#4	#5	#6
Day of onset post HSCT	1	5	40	20	7	15

CSA levels (ng/ml)	69	250	194	179	177	150

Mg levels (mEq/l)	0.88	0.86	0.95	0.72	0.85	1.03

Cholesterol and tryglicerides levels (mg/dl)	123/82	152/64	125/137	195/139	136/162	177/79

Prodromes	headache & vomiting	headache & HTN	HTN	HTN	HTN	HTN & blurred vision

Neurological signs	gen seiz	confusion	gen seiz	gen seiz	gen seiz	blurred vision

Acute treatment	LOR P PB	mannitol*	LOR P	LOR P	LOR P	mannitol*

Chronic treatment	P PB	none	P	P	P	P CLO

Outcome (day last follow-up	Alive & well (+1065)	Alive & well (+1059)	Alive & well (+1005)	Alive & well (+760)	Died, grade IV GVHD (+60)	Alive & well (+320)

Mg = magnesium

HTN = arterial hypertension

gen seiz = generalized seizures

LOR = lorazepam 0.05 mg/kg/dose iv

P = phenitoyn 15 mg/kg/dose iv for acute treatment

phenitoyn 7 mg/kg/day iv for maintenance treatment

PB = Phenobarbital 7 mg/kg/day iv

* 0.5 g/kg/dose iv

CLO = oral clonazepam 0.05 mg/kg/day

### Neuroradiological and electrophysiological findings

**Figure 1:**
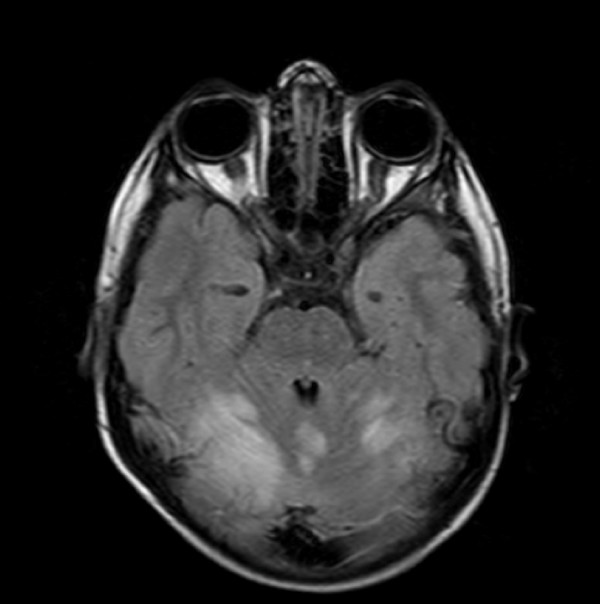
**MRI-flair axial study of the brain (patient #1) showing cortical and subcortical spots with a mild mass effect, with major spreading in the cerebellum (right hemisphere > left hemisphere)**.

**Figure 2:**
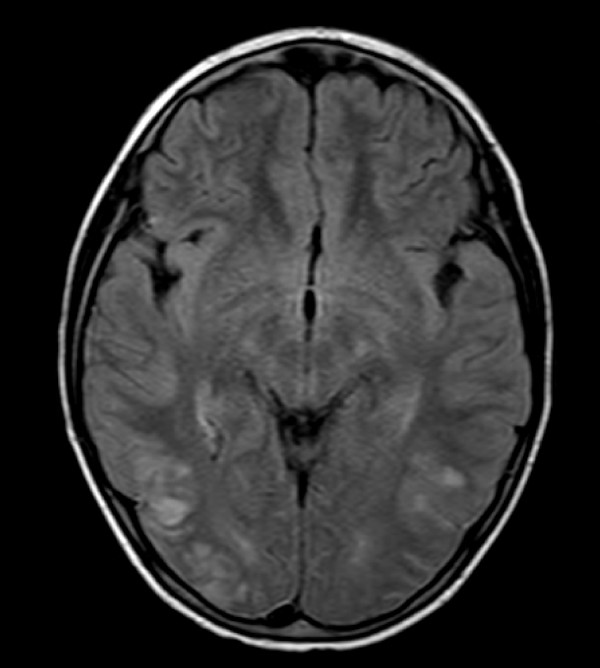
**MRI-flair axial study of the brain (patient #3) showing multiple cortical and subcortical spots involving both occipital hemispheres**.

Neuroradiological and electrophysiological studies were performed within 24 hours from neurological symptoms appearance. A CT scan performed in patient #5 because MRI was not immediately available showed a minimal subdural haemorrhagic layer compatible with post epileptic trauma. MRI was performed in all patients. In 5/6 patients, MRI showed a symmetric involvement of the posterior portions of the cerebral hemispheres, while one case showed abnormal signals hyperintense in T2 and Flair images in the cerebellar hemispheres, involving predominantly the white matter but also the cortex (figure 1, 2). In 5 out of the 6 patients studied, a moderate gyral swelling was present. On diffusion weighted images these lesions had an increase diffusion (hyperintens sygnal on apparent diffusion coefficient maps) consistent with vasogenic oedema.

**Figure 3:**
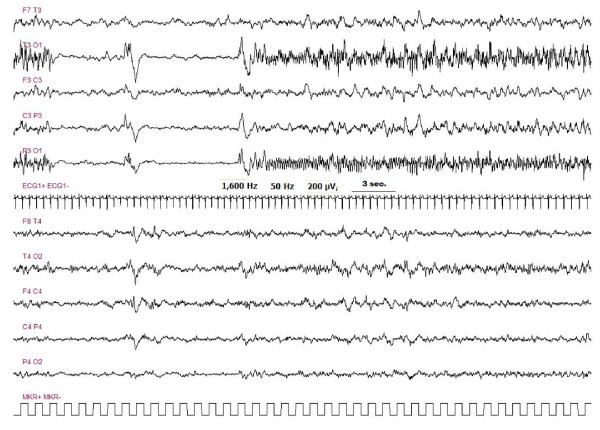
**Pt # 1 EEG pattern of a seizure, with left occipital onset, during status epilepticus**. Each seizure was close to the next one, with an inter critical activity lasting 10-30 seconds. In the majority of cases seizures were not related to an evident modification of the clinical status.

**Figure 4:**
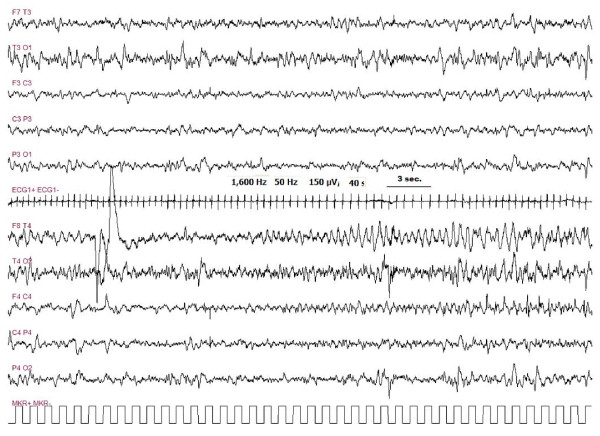
**Pt # 5 EEG pattern of a seizure, with right fronto-temporal onset**. Seizures were repeated every 20-40 minutes. During the seizures no clear modification of the clinical status was observed.

**Figure 5:**
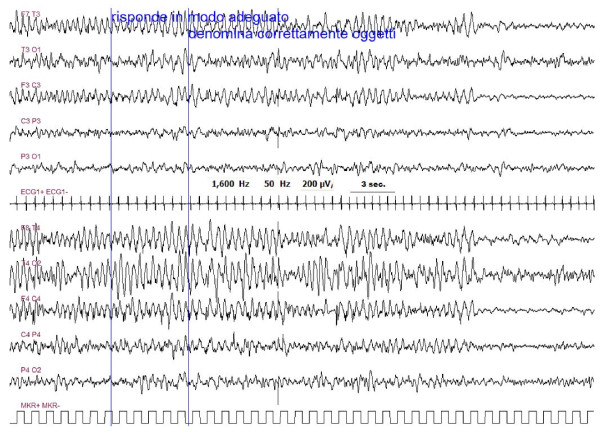
**Same patient as figure 4**. Ending of the seizure with contra-lateral diffusion of the epileptic abnormalities. During this phase the patient was able to answer questions and could name objects correctly. No motor epileptic sign of seizure were present.

In five out of 6 children, it was possible to perform an EEG at the time of the neurological symptoms onset. EEG of three out of five patients showed a status epilepticus (Figure 3, 4, 5). The other two patients showed, respectively, a focal delta activity and focal epileptiform discharges. EEGs were repeated every 2-3 days in the acute phase in order to adjust the anticonvulsive therapy.

### Treatment

On occurrence of neurotoxicity, CSA was immediately withdrawn. After 2 days of calcineurin-inhibitor washout, tacrolimus alone (n = 3) or tacrolimus and mycophenolate mofetile (n = 3) (according to the individual GvHD risk) were introduced with tacrolimus level monitoring. Neurological symptoms resolved within 2-3 days following CSA withdrawal and did not re-occur during tacrolimus treatment. Intravenous lorazepam was immediately administered at seizures unset, followed by anticonvulsive treatment with phenitoyn. Patient #1 (SCD) required association with phenobarbital to control generalized seizures (table 3). Intravenous mannitol was used successfully in patient #2 and #6 to treat confusion, vomiting, headache and blurred vision related to endocranial hypertension. Anticonvulsive therapy was tapered and stopped after normalization of EEG findings. In patient #6 phenitoyn was withdrawn after 3 weeks of treatment because of the onset of a severe hepatic-toxicity (elevation of liver enzymes and bilirubin) in the presence of a liver biopsy suggestive of drug-related injury. Liver function normalized following phenitoyn discontinuation. Anticonvulsive therapy was continued with clonazepam only, without seizure recurrence.

Outcome

Neuroradiological monitoring was performed by MRI every 7-14 days until complete remission of the diffuse signal alterations, which occurred after a median of 15 days (range 12-90 days) from CSA withdrawal. Non epileptiform EEG abnormalities were still detectable in all patients in the 15 days after the onset of symptoms. The complete normalization of EEG occurred after a median of 120 days (range 60-140 days). Five patients are alive and symptoms-free at a median follow up of 1005 days (range 320-1065) without neurological sequelae, while one patient died on day 60 for grade IV GvHD, without neurological symptoms.

## Discussion

CSA neurotoxicity is a well recognized complication observed after HSCT. Incidence of neurotoxicity in patients receiving allogeneic HSCT varies according to the underlying disease[6]. In the paediatric age incidence in haematological malignancies ranges between 4% and 11% [3,12]. The incidence is higher in non malignant disorders, although very few data have been published. In patients affected by haemoglobinopaties, a single published series suggests a very high incidence of CSA related neurotoxicity (28.8%)[7]. We describe a large and homogenous series of children with haemoglobinopaties who experienced a 9% incidence of CSA neurotoxicity following HSCT. Our data confirm previous findings on increased risk of CSA related severe neurotoxicity in patients with non malignancies, but suggests that, up to date, early diagnosis and intervention may ameliorate the incidence and severity. Patients with hemoglobinopaties may be at increased risk of neutoxicity due to impaired liver function secondary to iron overload as consequence of irregular iron chelation and blood-born viral infection. Both risk factors were heavily present in our series and the series reported by Erer et al[7]. CSA is metabolized in the liver as the drug is converted by the iso-enzymes of hepatic cytochrome P450. Impaired hepatic function may thus affect CSA metabolism triggering neurotoxicity. Another factor which may have influenced the onset of seizures is the use of iv busulfan in the preparative regimen. It has been reported that busulfan may induce seizures at the time of administration, and this may play a role in the subsequent appearance of neurotoxicity due to CSA. However, our policy of therapeutic dose adjustment and seizure prophylaxis during administration did not result in any immediate neurological toxicity.

Moreover, our results suggest that early patients' monitoring by neuro-radiological and electrophysiological studies and improved awareness of prodromes can reduce the incidence and long-term consequences of these events. Among the prodromes, arterial hypertension was the most premature and should therefore routinely evaluated and promptly intervened on. In our series, prompt discontinuation of CSA resulted in good prognosis with no neurological sequels. As alternative GvHD prophylaxis, we opted for tacrolimus. This calcineurin inhibitor, although similar to CSA in mechanism and metabolism, did not produce neurological side effects in this series.

## Conclusions

Our results confirm the high incidence of CSA related neurotoxicity in patients with non malignant disorders undergoing HSCT. This could be explained by their impaired organ functions due to the underlying disease or need for higher CSA doses. The awareness of prodromes of CSA related neurotoxicity (arterial hypertension, headache and visual disturbances) and early recognition with electrophysiological and imaging strategies is very important for prompt intervention on these patients, in order to reduce the risk of CSA related neurological events, in particular irreversible sequels. Our experience confirms that the gold standard approach to evaluate CSA related brain lesions is MRI, highly sensitive to detect the posterior diffuse alterations consistent with vasogenic oedema typical for PRES, and that CSA withdrawal should be prompt to prevent irreversible sequels.

## Abbreviations

CSA: cyclosporine A; HSCT: haematopoietic stem cell transplantation; PRES: posterior reversible encephalopathy syndrome; CNS: central nervous system; MRI: magnetic resonance imaging; EEG: electroencephalography; GvHD: graft versus host disease; SCD: sickle cell disease; B-thal: beta thalassemia.

## Competing interests

The authors declare that they have no competing interests.

## Authors' contributions

AN and BC participated in the data collection and interpretations of results and the preparation of the manuscript, FM, GF performed the electrophysiological tests and helped with the therapeutic decisions, SM, RC, AB, IF and EB participated in the study design and helped to draft the manuscript, CB and PV provided the neuroradiological MRI findings, RF helped with intensive care, FC and MGR helped in the interpretation of results and revised the manuscript. All authors read and approved the final manuscript.
